# Notes and News

**Published:** 1905-07-15

**Authors:** 


### Notes and News.

A special shilling fund has been instituted by the British Home and Hospital for Incurables, in order to pay off a loan from the bankers.

The late Sir John Willox, of Liverpool, has bequeathed £10,000 each to the Royal Infirmary, the Royal Southern and the Stanley Hospitals Liverpool.

The late Mrs. Eleanor Moses, of Carlisle, has left the majority of her estate, amounting to £28,600, to be divided between eight institutions, amongst which are the Cumberland Infirmary, the Cumberland and Carlisle Sanatorium for Consumption, and the Victoria Hospital at Maryport.

Mr. Edmund Owen, St. Mary's Hospital, and Mr. R. Godlee, University College Hospital, have been re-elected members of the Council of the Royal College of Surgeons, and Mr. C. Goldny Bird, Guy's Hospital, and Mr. W. Harrison Cripps, St. Bartholomew's Hospital, have been elected members of the Council.

The will of the late Mr. Wyndham Francis Cook, of St. Paul's Churchyard and Manchester, provides for a gift of £25,000 to the central Hospital funds, of which the Sunday Fund has already received £12,500, as we announced last week, and the remaining moiety has been received by King Edward's Hospital Fund. Mr. Cook further bequeathed £1,000 to the Royal Ophthalmic Hospital, City Road, E.C.

The distribution of certificates and prizes took place at King's College on Wednesday afternoon. The past year has been financially prosperous, owing to the increased Treasury grant received. A further increase is promised. A first step has been made towards concentration of medical studies in the earlier subjects, an arrangement made by which students of Westminster Hospital are to attend King's College. Dr. Gow distributed the awards, mentioning the fact that just forty years ago he had made his *début* there as a small boy at the bottom of the school.

Professor Koch announces that he has dis-covered, in the tsetse fly of trypanosoma, the bacillus of tsetse fever.

The National League for Physical Education and Improvement held a meeting following that at the Mansion House on July 3rd, and agreed to the federation with the League of those societies concerned in questions of health, the members of which formed part of the Conference.

The Committee of the Mary Wardell Convalescent Home, Stanmore, are issuing an earnest appeal for at least £1,000. The institution provides a home for patients recovering from scarlet and other infectious illnesses from all parts of the kingdom, for whom there is little accommodation available. The twenty-fifth anniversary of the Home was celebrated by a meeting at the Mansion House on July 13th, which it is to be hoped will result in a ready response to the appeal.

There are few institutions which confer a more definite benefit upon the afflicted members of the community than the Association for the Oral Instruction of the Deaf and Dumb, which aims at providing skilled teachers. Those who attended the annual meeting last week were able to witness the surprising successful results of the teaching upon the pupils of the Association. The dumb are literally taught to speak, and the deaf to interpret speech by observation of the lips of speakers. The Association is in need of support, but we hope that the striking demonstration of a useful work well carried out will result in ample funds being forthcoming.

At the last meeting of the Metropolitan Asylums Board, Professor Smith observed that the question of the hospital treatment of scarlet fever and diphtheria cases was rapidly becoming one of great national importance. Imputations had been made that the discharged cases had been sent home in such a condition that they were infectious. He thought there was no doubt that that had been the case, but he was quite convinced that the evil was not to be met by prolonged detention in hospital. Then the question of complications was one of very great importance. It seemed something like a scandal that a child sent into the hospital with scarlet fever should contract diphtheria when in the wards. If the hospitals were not managed in a way to prevent the spread of disease the question arose whether the public was justified in incurring the enormous expense. The questions raised were of such great importance that nothing short of a Royal Commission to go fully into the matter would put the subject on a satisfactory footing. He hoped that recommendations would be made to the Local Government Board to that effect. The committee's recommendation was adopted. As the result of observations and investigations the Board are about to experiment in one of their hospitals by trying a treatment of complete isolation for scarlet fever patients during the whole term of their detention. The result in the direction of return cases will then be carefully watched.

